# Severe tremors induced by tiletamine e-cigarette and alcohol use: a case report

**DOI:** 10.3389/fpsyt.2025.1537822

**Published:** 2025-04-03

**Authors:** Bojie Zhou, Shanghao Yang, Xiafeng Zhou, Qian Chen, Ewen Tu, Bo Zhang, Li Shi, Xuhui Zhou

**Affiliations:** ^1^ The School of Clinical Medicine, Hunan University of Chinese Medicine, Changsha, China; ^2^ Department of Addiction Medicine, Hunan Institute of Mental Health, The Second People’s Hospital of Hunan Province (Brain Hospital of Hunan Province), Changsha, China; ^3^ Department of Psychiatry, Dongguan Seventh People’s Hospital, Dongguan, China; ^4^ Department of Neurology, The Second People’s Hospital of Hunan Province (Brain Hospital of Hunan Province), Changsha, China; ^5^ Hunan Society of Traditional Chinese Medicine and Integrated Traditional Chinese and Western Medicine, Changsha, China

**Keywords:** tiletamine, alcohol, primidone, phencyclidine use disorder, polydrug use, withdrawal, e-cigarettes, case report

## Abstract

**Background and objectives:**

Polydrug use has caused serious harm to public health, especially involving novel psychoactive substances. Tiletamine, an N-methyl-D-aspartate receptor (NMDAR) antagonist commonly used as a veterinary anesthetic, has recently emerged in China as an additive in e-cigarettes. However, the long-term impacts of tiletamine and its combined use with other substances remain poorly understood. This case report aims to provide further insight into the clinical manifestations and treatment of tiletamine abuse, particularly focusing on the tremors induced by polydrug use.

**Case presentation:**

The patient had five years of intermittent alcohol use and five months of etomidate abuse. After combining tiletamine for two months, he was repeatedly hospitalized due to coarse tremors, poor sleep and appetite. Based on his substance use pattern and related outcomes, he was diagnosed with phencyclidine use disorder. Initially, intravenous diazepam (20 mg/day) effectively alleviated the tremors. During the second hospitalization, the same dose took longer to take effect, and by the third hospitalization, the dose was increased to 30 mg/day without reducing the tremors. Therefore, primidone was added and gradually titrated to 50 mg/day. The patient’s tremors began to improve by the eighth day and significantly diminished by the tenth day. As we gradually replaced diazepam with lorazepam, the patient insisted on discharge.

**Conclusions:**

Polydrug users, particularly those using NMDAR antagonists and gamma-aminobutyric acid type A receptor (GABA-AR) agonists, may be at increased risk of developing tiletamine dependence, with more severe consequences due to cross-addiction. The combination of alcohol and tiletamine could exacerbate neuroexcitotoxicity during withdrawal, potentially contributing to severe tremors. The successful management of tremors with a combination of neuroinhibitory therapies suggested an effective strategy for complex cases. Further studies are needed to better understand the long-term impacts and risks of tiletamine dependence.

## 1 Introduction

In recent years, there has been a significant rise in the incidence of polydrug use, varying from 3.7%–49.7% in different western countries (1–3), elevating it to a critical public health concern. The primary consequences encompass a heightened risk of accidents or injuries, drug overdose, and fatalities (4). Polydrug use is defined as the concurrent or sequential use of multiple legal or illegal addictive substances (4). Among these, alcohol holds a prominent position due to its widespread availability; an Italian study analyzing outpatient cases revealed that 60% of abuse instances involve alcohol (5). In China, with the rapid economic development, alcohol consumption has been increasing, which has raised concerns about alcohol-related issues (6). Long-term alcoholics often experience postural tremors when they reduce or stop drinking (7). In some cases, even without complete withdrawal, long-term drinking can lead to persistent tremors, which may be related to the chronic brain damage along with other neurological issues (8). The combination of alcohol with other drugs can exacerbate adverse effects such as withdrawal symptoms, cardiovascular disease, liver damage, and behavioral abnormalities (9).

Recently, the stringent regulation of traditional drugs has prompted illegal manufacturers to explore novel psychoactive substances (NPS) as substitutes for illicit drugs. NPS that are most closely linked to psychiatric consequences include synthetic cannabinoids, novel synthetic opioids, and ketamine analogs (10). The proliferation of these substances has been facilitated by the shift from street-level drug markets to online platforms, making them more accessible to a broader audience (11). Despite efforts to control their spread, NPS continue to pose a significant challenge due to their ever-evolving nature and the difficulty in regulating them effectively (12). In China, the drug abuse pattern has also transitioned from traditional opiates to a combination of new synthetic drugs, medical anesthetics, and psychoactive substances (13). Since 2022, reports have emerged concerning the abuse of etomidate e-cigarettes in China. Due to its severe addictive potential and harmful effects, etomidate was officially regulated in China as of October 1st, 2023. Subsequently, the veterinary anesthetic tiletamine has gradually emerged as a novel drug substitute, also sold and consumed in e-cigarette form, posing a significant threat to public health and safety.

Tiletamine is a non-competitive NMDA receptor antagonist with structural and pharmacological similarities to phencyclidine (PCP) and ketamine, known for its potent analgesic and dissociative anesthetic properties (14). It is often combined with the GABA-A receptor agonist zolazepam, which provides mild sedation and effective muscle relaxation (14). Tiletamine and zolazepam are combined in a 1:1 ratio to form the compound anesthetic Telazol (Zoetis Inc., New Jersey, USA). In Asia, this anesthetic is commonly known as Zoletil (Virbac, Lyon, France). Telazol abuse has been reported since 1999 (15). Initially confined to veterinary workers (15, 16), Telazol use has expanded to include the general public (17). The primary routes of abuse include intravenous bolus (18), intramuscular injection (19), and nasal inhalation (17). Most reported cases involve recreational use, with two resulting in fatalities (15, 16), and one in a failed suicide attempt (18). Due to its societal harm, tiletamine has been classified as a controlled substance in countries including South Korea and the United States (20).

Although tiletamine is gaining popularity in other countries, its consequences remain insufficiently explored. In China, where tiletamine use is still emerging, there is no medical evidence documenting its abuse or addiction. Its long-term effects and potential dependence are not well understood, nor are the outcomes of co-use with e-cigarettes and alcohol, presenting significant challenges to treatment services and regulatory oversight. These developments highlight the urgent need for more case studies to identify the clinical symptoms and effective recovery strategies to mitigate the public health impact of tiletamine. A key concern is the movement disorders associated with tiletamine (Telazol or Zoletil) use (21, 22), which are primarily manifested as tremors in this case. Herein, we report a case of severe tremors resulting from polydrug use, primarily involving alcohol and tiletamine e-cigarettes, aiming to provide further insight into the clinical manifestations and treatment of tiletamine abuse.

## 2 Case

A 40-year-old male presented with recurrent limb tremors, poor appetite, and sleep disturbances, leading to repeated hospitalizations over three months. He has a long history of substance use, beginning in 2005 with ketamine (4 years), followed by heroin (3 years), and methamphetamine (6 years). After each substance, he underwent compulsory detoxification for one year. In 2019, following his last detoxification, he intermittently consumed high-proof alcohol (400–500 ml per occasion), primarily during social gatherings. In June 2023, he began using etomidate-containing e-cigarettes, which led to a car accident due to hazardous driving. He discontinued etomidate after the accident but switched to tiletamine-containing e-cigarettes in October 2023, escalating his use from a few times a week to daily (from two to six cartridges). Despite experiencing euphoria initially, he later developed depressive moods and suicidal thoughts. His alcohol consumption continued, but he did not report withdrawal symptoms. Key milestones in the patient’s substance use history were illustrated in **Table 1**.

**Table 1:** Key milestones in the patient’s substance use history.

Duration	Abusing substance	Dose	Adverse consequences	Summaries from history and examination	Diagnosis/treatment
2005–2008	Ketamine	The patient could not recall	ns	Dissociative symptoms, visual hallucinations, depressive moods	Phencyclidine use disorder/mandatory detoxification in a rehabilitation center for one year
2009–2011	Heroin	The patient could not recall	ns	The patient could not recall	Opioid use disorder/mandatory detoxification in a rehabilitation center for one year
2012–2017	Methamphetamine	The patient could not recall	ns	The patient could not recall	Methamphetamine use disorder /mandatory detoxification in a rehabilitation center for one year
2018–present	High-proof liquor	400–500 ml per occasion, primarily during social gatherings, without a regular pattern, every few days	ns	ns	ns
June 2023–September 2023	Etomidate e-cigarettes	Two cartridges per occasion, every few weeks, with the exact dosage per cartridge unknown	A car accident due to hazardous driving after using etomidate	Dizziness, confusion, inability to stand, and falls after using etomidate	ns/the patient discontinued etomidate use on his own without experiencing any adverse effects after withdrawal
October 2023–February 2024	Tiletamine e-cigarettes	Increased from several times a week to daily within a month, with each session escalating from two to eight cartridges per day, with the exact dosage per cartridge unknown	A suicidal attempt	Dissociative symptoms, visual hallucinations, depressive moods, suicidal thoughts and attempts, poor appetite, sleep disturbance, unsteady gait, limb tremors, head tremors, voice tremors, dysarthria, irritability, anxiety, chest tightness, nausea, dizziness	Phencyclidine use disorder/the patients’ treatment were detailed in **Table 3**

On November 28, 2023, the patient developed involuntary tremors in both upper limbs, which worsened with activity. He was prescribed diazepam(10mg/day), which alleviated the tremors, but his symptoms returned after resuming tiletamine use. He was hospitalized on December 4, 2023, and received intravenous diazepam(20mg/day), after which he improved and requested discharge. Upon discharge, he resumed alcohol consumption, which further reduced the tremors. He relapsed to tiletamine on January 21, 2024, and increased his dosage (eight cartridges per day). By January 27, 2024, he reduced his alcohol intake by half. On February 5, 2024, he experienced worsening tremors and was admitted to our hospital, where he received intravenous diazepam(20mg/day) with significant improvement. However, after discharge, he relapsed and resumed previous consumption levels without drinking alcohol. On February 20, 2024, he was readmitted after his tremors worsened due to abrupt discontinuation of tiletamine, along with chest tightness, nausea, and dizziness. A review of his medical history revealed two prior admissions for limb tremors, with details of diagnoses and interventions summarized in **Table 2**.

**Table 2:** Admission timeline.

Date of admission	Tiletamine use	Alcohol use	Days at hospital/successive days of tiletamine use	Summaries from history and examination	Diagnosis/treatment	Hamilton Anxiety Rating Scale
December 4, 2023	Increased from two to six cartridges per day (from a few times a week to daily)	400–500 ml of high-proof liquor per occasion, every a few days	2/33	Dissociative symptoms, visual hallucinations, depressive moods, suicidal thoughts and attempts, poor appetite, sleep disturbance, limb tremors, unsteady gait,	Phencyclidine use disorder/ intravenous diazepam 20 mg/day, intravenous fluid 1000 ml/day, sodium valproate 0.5 g/day	15
February 5, 2024	Increased from six to eight cartridges per day	Reduced by half (150–200 ml of high-proof liquor per occasion) 7 days before admission	4/14	Poor appetite, sleep disturbance, limb tremors, unsteady gait, irritability	Phencyclidine use disorder/intravenous diazepam 20 mg/day, intravenous fluid 1000 ml/day, sodium valproate 0.5 g/day	14
February 20, 2024	Eight cartridges per day	ns	12/9	Poor appetite, Sleep disturbance, unsteady gait, limb tremors, head tremors, voice tremors, dysarthria irritability, anxiety, chest tightness, nausea, dizziness	Phencyclidine use disorder/intravenous diazepam 30–20 mg/day, lorazepam 2 mg/day, intravenous fluid 1000 ml/day, sodium valproate 0.5–1.0 g/day, olanzapine 5–10 mg/night, primidone 25–50 mg/day	25

**Figure 1:**
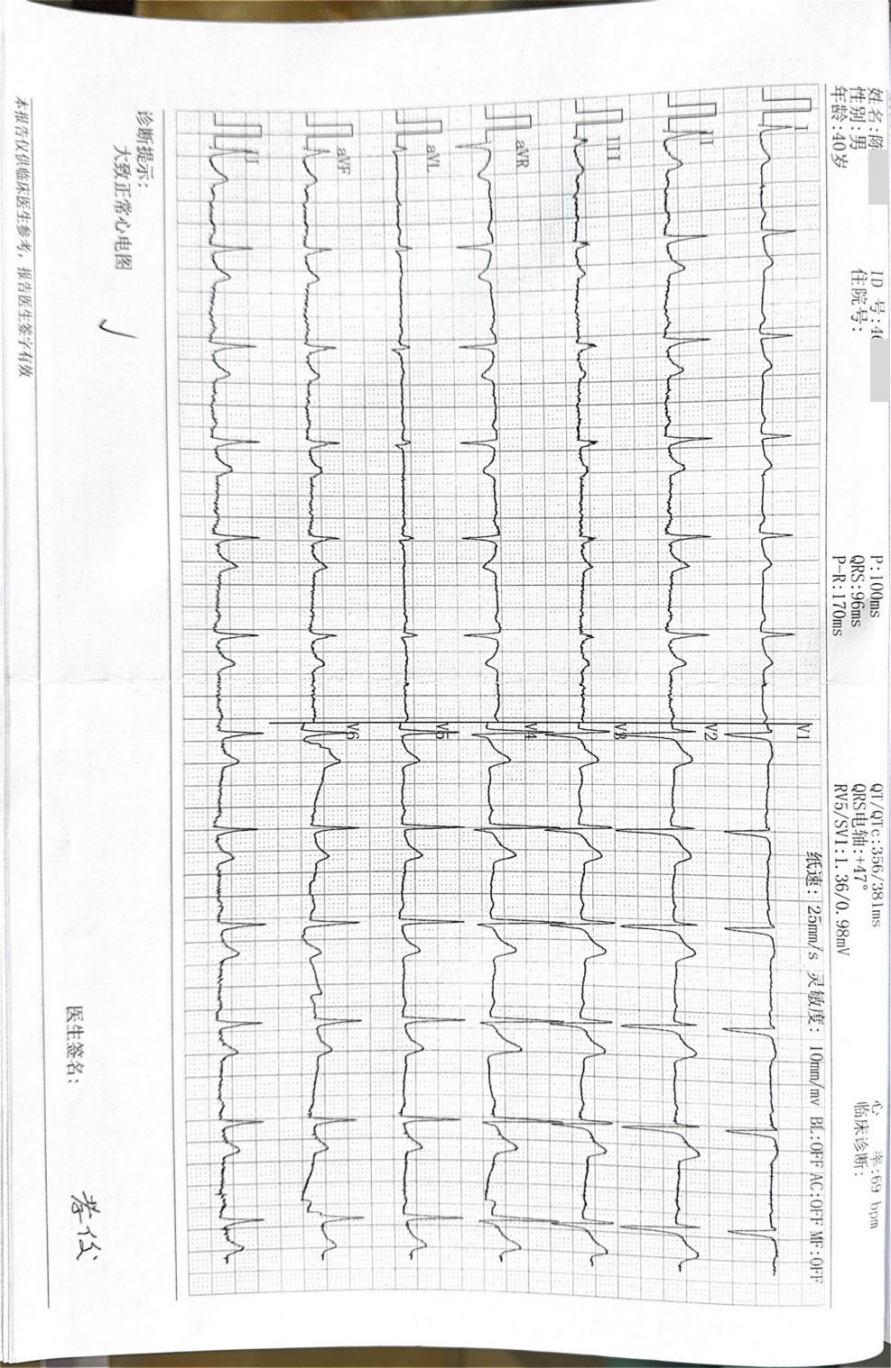
Electrocardiogram (ECG) images. The electrocardiogram showed a generally normal rhythm with a heart rate of 69 beats per minute.

**Figure 2:**
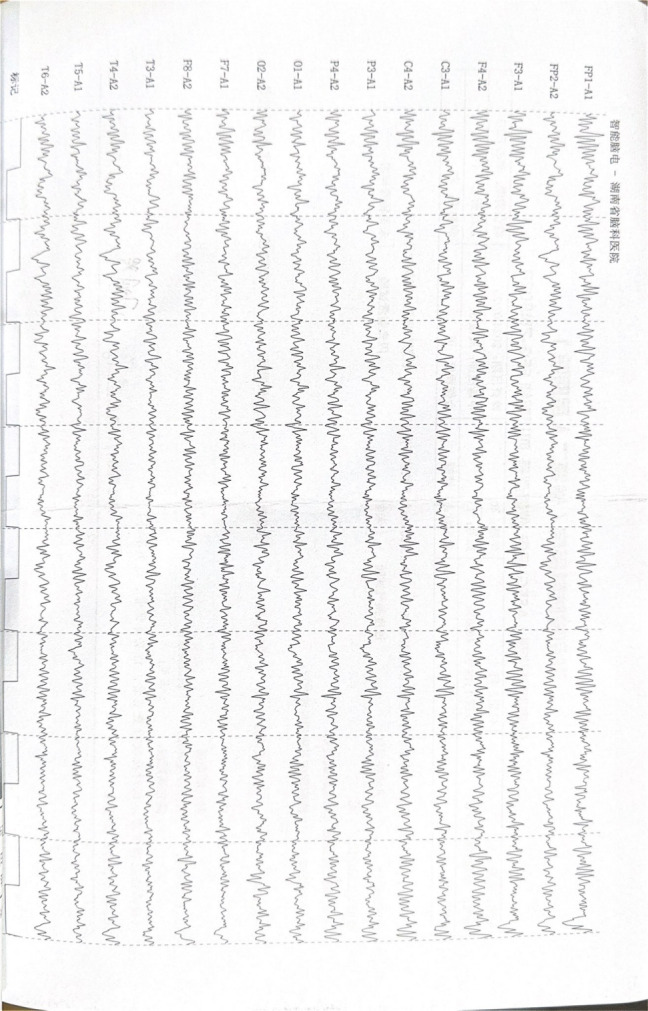
Electroencephalogram (EEG) images. The electroencephalogram showed abnormal findings with mild background activity. The primary rhythm observed during quiet wakefulness with eyes closed was a low-amplitude (5–10 μV), 15–20 Hz rhythmic activity, which appeared somewhat irregular. Bilateral symmetry was present.

The patient’ s vital signs were normal upon first two admission. On the third hospitalization, he was clear with a blood pressure of 128/93 mmHg, pulse rate of 59 beats per minute, respiration rate of 20 breaths per minute, and body temperature of 36.9°C. The observable tremors in four limbs, about 5 centimeters in amplitude, intensified with voluntary movement; however, his muscle tone remained normal. He also exhibited an unsteady gait, head tremor, voice tremor, slurred articulation and variable voice intensity. Increased irritability was noted, but hallucinations and delusions were denied. Recent laboratory tests showed no abnormalities in routine blood tests, coagulation profiles, blood ammonia, electrolytes, liver and kidney function, cardiac enzymes, blood glucose, lipid profiles, or thyroid function. Urinalysis was negative for benzodiazepines, morphine, ketamine, ecstasy, methamphetamine, and buprenorphine. The electrocardiogram (ECG) showed a normal rhythm with a heart rate of 69 beats per minute. Brain magnetic resonance imaging (MRI) revealed a cavernous hemangioma in the right basal ganglia, and the electroencephalogram (EEG) showed a mildly abnormal fast-wave background. The images of the ECG and EEG are presented in **Figures 1**, **2** respectively.

Brain MRI showed nonspecific abnormalities in the basal ganglia, which could not suggest any organic brain disorder or explain the onset of the tremors. The patient’s alcohol consumption pattern did not meet the criteria for alcohol dependence, and he did not exhibit symptoms such as sweating, elevated blood pressure, or tachycardia, thereby ruling out alcohol withdrawal as the cause of the tremors. Although we were unable to detect tiletamine using our current techniques, considering that the patient exhibited a pattern of self-reported tiletamine use (structure and potency similar to phencyclidine), characterized by high-dose consumption, intense cravings, increased tolerance, and recurrent physical and interpersonal issues meeting over two criteria from the DSM-5, we diagnosed the patient with phencyclidine use disorder.

To manage his tremors and associated symptoms, pharmacologic treatments outlined in **Table 3** were administered. He received intravenous diazepam at 30 mg/day. Due to irritability, poor sleep and appetite, oral sodium valproate (0.25 g twice daily) and olanzapine (5 mg nightly) were started. Additionally, 500 ml of intravenous fluids were given twice daily to maintain physiological stability. As his appetite improved, intravenous fluid administration was gradually discontinued. By the sixth day, his sleep, anxiety and irritability had significantly improved, with sodium valproate at 1 g/day, olanzapine at 10 mg/night and the Hamilton Anxiety Rating Scale score dropping from 25 to 10. However, tremors showed no improvement (5 centimeters in amplitude). Consequently, primidone was initiated at 25 mg nightly and gradually increased to 50 mg/day. By the eighth day, tremors began to subside (3 centimeters in amplitude), with marked improvement by the tenth day (1 centimeters in amplitude). During this period, diazepam was gradually replaced with oral lorazepam. By the twelfth day, the patient was able to walk normally, though mild tremors persisted in both upper limbs. The following day, the patient requested discharge and declined further medication. No significant adverse drug reactions occurred throughout treatment.

**Table 3:** Detailed medication administration during the third hospitalization.

	Day 1	Day 2	Day 3	Day 4	Day 5	Day 6
Diazepam	10 mg q 8 hours IV	10 mg q 8 hours IV	10 mg q 8 hours IV	10 mg q 8 hours IV	10 mg q 8 hours IV	10 mg q 8 hours IV
Sodium Valproate	0.25 g QD 0.25 g QN PO	0.25 g QD 0.25 g QN PO	0.25 g QD 0.25 g QN PO	0.5 g QD 0.5 g QN PO	0.5 g QD 0.5 g QN PO	0.5 g QD 0.5 g QN PO
Intravenous Fluid	500 ml Bid IV	500 ml Bid IV	500 ml QD IV	500 ml QD IV	Discontinued	
Olanzapine		5 mg QN PO	5 mg QN PO	5 mg QN PO	10 mg QN PO	10 mg QN PO
Primidone						25 mg QN PO

	Day 7	Day 8	Day 9	Day 10	Day 11	Day 12
Diazepam	10 mg q 8 hours IV	10 mg q 8 hours IV	10 mg q 12 hours IV	10 mg q 12 hours IV	10 mg q 12 hours IV	10 mg q 12 hours IV
Sodium Valproate	0.5 g QD 0.5 g QN PO	0.5 g QD 0.5 g QN PO	0.5 g QD 0.5 g QN PO	0.5 g QD 0.5 g QN PO	0.5 g QD 0.5 g QN PO	0.5 g QD 0.5 g QN PO
Olanzapine	10 mg QN PO	10 mg QN PO	10 mg QN PO	10 mg QN PO	10 mg QN PO	10 mg QN PO
Primidone	25 mg QN PO	25 mg QN PO	50 mg QN PO	50 mg QN PO	50 mg QN PO	50 mg QN PO
Lorazepam			2 mg QD PO	2 mg QD PO	2 mg QD PO	2 mg QD PO

The patient requested discharge on day 13 without receiving any medication.

In the six-month follow-up conducted by phone, the patient reported no use of tiletamine e-cigarettes or tremors, while maintaining his previous pattern of alcohol consumption.

## 3 Discussion

To the best of our knowledge, this is the first documented case of polydrug use involving alcohol and e-cigarettes containing etomidate and tiletamine. The existing literature lacks detailed descriptions of tiletamine dependence and effective treatment recommendations. Compared to previously reported cases of tiletamine or Zoletil abuse, the primary symptom in this case was severe limb tremors. Consequently, our treatment focused primarily on addressing the tremors and successfully managed the acute withdrawal phase.

Animal studies suggest that tiletamine, when used as a sole anesthetic, can induce muscle rigidity and seizures (23), similar to the effects observed in humans after a single large dose (17). Acute tiletamine poisoning is often fatal and can result in changes in mental status, nystagmus, cardiac dysfunction, and metabolic disruption (15, 16, 24, 25), while chronic use has been associated with psychoses and behavioral abnormalities (19), with rare reports of movement disorders. For instance, Lee reported a 35-year-old man who intermittently used heroin and inhaled Telazol to reduce heroin dosage, gradually developing choreatic movements after two weeks, which were alleviated with clonazepam after two weeks (21). Similarly, a 30-year-old male who consumed 2,250 mg of Zoletil over 9 days developed involuntary tremors of the lower jaw, failing eyesight, and sialorrhea one day after discontinuing the injection. The symptoms self-resolved without any intervention (22). In our case, tremors in four limbs were consistently observed in relation to tiletamine and alcohol use.

The patient’s tremors were initially mild and gradually worsened. Similar symptoms have been reported in ketamine users. In a 10-year cross-sectional study in Italy involving 7,897 cases of illegal substance use, 74 cases were ketamine-related. Tremors accounted for 6.8% of the reasons ketamine users sought emergency department care, and these tremors were typically mild and transient (26). This case, however, presented with intentional tremors, dysarthria, and ataxia, suggesting a cerebellar dysfunction. Interestingly, alcohol alleviated these tremors, similar to essential tremor (ET), where alcohol activates GABA-A receptors in cerebellar granule cells, reducing tremors (27–29). Additionally, repeated ketamine administration has been shown to upregulate NMDAR expression in the frontal cortex while downregulating GABA-AR expression, paralleling the modulation observed in chronic alcohol consumption models (30). Furthermore, the increase in its subunit GRN1 and the resultant neurotoxicity are dependent on both the duration and dosage of ketamine exposure (30). Thus, we hypothesize that repeated high-dose tiletamine use may cause neurotoxicity in cerebellar granule cells, increasing excitotoxicity during withdrawal. Alcohol, a GABA-A agonist, could counterbalance this excitotoxicity. However, with prolonged use, the balance between inhibitory and excitatory neurotransmission may be reestablished and then disrupted by abrupt cessation or dose reduction of either substance, leading to tremors.

However, the patient’s tremors were more severe than those induced by either ketamine or alcohol withdrawal, suggesting that polydrug use may have contributed to their intensity. The combination of alcohol and tiletamine likely resulted in synergistic neuroinhibition, heightening glutamatergic activity and excitotoxicity. Previous reports have shown that tiletamine abusers are typically polydrug users with histories of NMDAR antagonist or GABAR agonist use (31). Animal studies have shown that prior exposure to alcohol significantly enhances Zoletil place preference and self-administration, highlighting the cross-addiction and compensatory effects between ethanol and NMDA antagonists (32, 33). This cross-addiction may lead to increased consumption of both substances at high doses, potentially explaining the heightened severity of tremors. Although direct human studies are limited, preclinical data suggest that alcohol and tiletamine may together produce even stronger neuroexcitotoxic effects, similar to those observed with the combination of ketamine and ethanol (34, 35). Given tiletamine’s potency and effective duration between PCP and ketamine in animals (36), it is reasonable to expect tiletamine might produce even stronger neurotoxic effects in humans.

Accordingly, the tremors likely resulted from an imbalance between the excitatory glutamatergic and inhibitory GABAergic systems, exacerbated by acute withdrawal. During the first two hospitalizations, intravenous diazepam was initially effective in controlling the tremors. However, as tolerance developed, diazepam became less effective over time, necessitating higher doses. Given that the tremors were likely linked to excitotoxicity, we administered sodium valproate to increase GABA levels and stabilize mood, in addition to primidone to alleviate the tremors. This combination led to significant improvement, suggesting that neuroinhibitory therapies could effectively address the patient’s symptoms.

In addition, while deaths related to vaping products containing synthetic cannabinoids have been reported (37), and some studies have suggested that harmful constituents in vaping aerosols may cause cardiopulmonary dysfunction (38), it remains unclear whether e-cigarettes, as a medium, contribute to the dependence and neurotoxicity of tiletamine. Given the widespread use of e-cigarettes and the lack of regulatory policies on tiletamine in China, an increase in tiletamine abuse and dependence via e-cigarettes is expected, along with a potential rise in the risks associated with polydrug use. It remains uncertain whether e-cigarette use of tiletamine is more toxic than traditional methods. Vigilance is needed regarding the concurrent use of tiletamine and GABA-A agonists.

This study has several limitations. Due to constraints in testing methods, we were unable to conduct drug testing for tiletamine or quantify its amount in the e-cigarette. Given that both alcohol and tiletamine can cause neurological damage, we did not explicitly assess peripheral nerve damage in this patient, nor did we investigate potential deficiencies in vitamin B1 or B12. These factors should be considered in future clinical practice. Despite the effectiveness of pharmacological treatments in managing symptoms, improving treatment adherence in patients with complex polysubstance abuse remains challenging. In this case, the patient failed to complete the detoxification treatment during all three hospitalizations, suggesting a lack of recognition of the dangers of polysubstance abuse and insufficient motivation for withdrawal. A multi-disciplinary approach, including psychological therapy, behavioral interventions, and support groups, is crucial but difficult to implement consistently in clinical practice. Future efforts may need to focus more on prevention, with regular health education, substance use assessments, and psychological evaluations for individuals with a history of polysubstance abuse. This proactive approach would help identify problematic behaviors, the risk of drug dependence, and any potential side effects, allowing for timely intervention and improving the long-term management of polysubstance abusers to prevent relapse.

## 4 Conclusion

Polysubstance abusers, particularly those using NMDA antagonists and GABA-A agonists, could be at increased risk of developing addiction to tiletamine. The concurrent use of these substances could lead to more severe consequences due to cross-addiction and their ability to counteract each other’s negative effects. Long-term alcohol use induces adaptive changes in NMDA and GABA receptors, leading to faster tolerance development, requiring higher doses of tiletamine to achieve desired effects and complicating withdrawal treatment. Combined use of alcohol and tiletamine may exacerbate neuroexcitotoxicity during withdrawal by upregulating glutamatergic and NMDA receptor activity, potentially contributing to severe tremors as seen in this case. The effectiveness of adding primidone in treating tremors suggests that a combination of neuroinhibitory therapies may more effectively manage the complex tremors induced by polydrug use involving tiletamine and GABA-A receptor agonists. Further studies on tiletamine are needed to better understand its effects and potential risks on humans.

## Data Availability

The original contributions presented in the study are included in the article/supplementary material. Further inquiries can be directed to the corresponding author.
